# Daptomycin Liposomes Exhibit Enhanced Activity against Staphylococci Biofilms Compared to Free Drug

**DOI:** 10.3390/pharmaceutics16040459

**Published:** 2024-03-26

**Authors:** Foteini Gkartziou, Maria Plota, Charikleia Kypraiou, Iti Gauttam, Fevronia Kolonitsiou, Pavlos Klepetsanis, Iris Spiliopoulou, Sophia G. Antimisiaris

**Affiliations:** 1 Department of Pharmacy, School of Health Sciences, University of Patras, 26504 Patras, Greece; klairi.kp@gmail.com (C.K.);; 2 Institute of Chemical Engineering Sciences, FORTH/ICE-HT, Platani, 26504 Patras, Greece; 3 Department of Microbiology, School of Medicine, University of Patras, 26504 Patras, Greece; plotamaria@yahoo.com (M.P.); kolonits@upatras.gr (F.K.); 4 National Reference Centre for Staphylococci, School of Medicine, University of Patras, 26504 Patras, Greece; spiliopl@upatras.gr

**Keywords:** daptomycin, liposomes, zeta-potential, bacteriostatic, biofilm, growth inhibition, MRSA, MRSE, integrity

## Abstract

The purpose of the present study was to investigate the anti-staphylococcal activity of liposomal daptomycin against four biofilm-producing *S. aureus* and *S. epidermidis* clinical strains, three of which are methicillin-resistant. Neutral and negatively charged daptomycin-loaded liposomes were prepared using three methods, namely, thin-film hydration (TFH), a dehydration–rehydration vesicle (DRV) method, and microfluidic mixing (MM); moreover, they were characterized for drug encapsulation (EE%), size distribution, zeta-potential, vesicle stability, drug release, and drug integrity. Interestingly, whilst drug loading in THF and DRV nanosized (by extrusion) vesicles was around 30–35, very low loading (~4%) was possible in MM vesicles, requiring further explanatory investigations. Liposomal encapsulation protected daptomycin from degradation and preserved its bioactivity. Biofilm mass (crystal violet, CV), biofilm viability (MTT), and growth curve (GC) assays evaluated the antimicrobial activity of neutral and negatively charged daptomycin-liposomes towards planktonic bacteria and biofilms. Neutral liposomes exhibited dramatically enhanced inhibition of bacterial growth (compared to the free drug) for all species studied, while negatively charged liposomes were totally inactive. Biofilm prevention and treatment studies revealed high antibiofilm activity of liposomal daptomycin. Neutral liposomes were more active for prevention and negative charge ones for treating established biofilms. Planktonic bacteria as well as the matured biofilms of low daptomycin-susceptible, methicillin-resistant *Staphylococcus aureus* (*MRSA*) and *Staphylococcus epidermidis* (*MRSE*) strains were almost completely eradicated by liposomal-daptomycin, indicating the need for their further exploration as antimicrobial therapeutics.

## 1. Introduction

Between the major unmet medical needs that currently represent serious public health threats, methicillin-resistant *Staphylococcus aureus* (MRSA) infections (which are associated with high mortality rates) and, particularly, medical device-associated infections (MDIs), are of major concern [1,2,3,4]. MDIs are caused by a patient’s microbiota flora having a biofilm-forming ability. *Staphylococcus epidermidis* and *S. aureus* bacteria are the most common aetiologic pathogens for such infection [5,6].

Biofilm formation initiates via the attachment of bacteria and proceeds through proliferation and maturation in a matrix consisting of extracellular polymeric substances; this biofilm/matrix finally functions as a barrier to antibiotics and to the host’s defense mechanisms [6]. Biofilm bacteria develop resistance due to the modification of their growth rate and other physiological functions and, additionally, because of the slow penetration of antimicrobials. Furthermore, biofilm matrices also block neutrophil attacks [7]. In addition to the biofilm aggregate-caused tolerance to antibiotics, most clinical strains of staphylococci are multi-resistant [7,8].

Daptomycin (Dapto) is an acidic cyclic lipopeptide antibiotic that, in the presence of calcium, forms oligomeric pores on membranes containing phosphatidylglycerol. It is clinically used against various Gram-positive bacteria such as *Staphylococcus aureus* and *Enterococcus* species, and, in addition to its potent antimicrobial activity [9], Dapto is known due to the unlikely development of daptomycin-resistant pathogens, which is attributed to its unique mechanism of action [9,10]. The Infectious Disease Society of America proposes Dapto for use in the therapy of enterococcal and staphylococcal infections of prosthetic joints [11]. Another advantage of Dapto concerning the treatment of MDIs is its known high anti-biofilm activity [12,13,14]. In one case, it has been reported that Dapto rapidly penetrates a Staphylococcus epidermidis biofilm [15]. It was also recently demonstrated in our laboratories that various Staphylococci strains showed lower MIC to Dapto, under biofilm-forming conditions, suggesting that Dapto is active in embedded cells [16]. Nevertheless, the development of some Dapto-insusceptible MRSA isolates has been reported [17,18], raising serious concerns and indicating the importance of developing novel strategies for antimicrobial therapeutics.

One such strategy currently being explored is the delivery of antibiotics or antimicrobials in general via nanomedicines [19,20,21]. For example, it has been reported that Dapto encapsulated in polymeric nanoparticles demonstrated higher activity towards established Staphylococci biofilms compared to the free drug [19].

As a nanomedicine type, liposomes were selected due to the numerous advantages that they possess as nanomedicines, given their high biocompatibility, ability to be loaded with high amounts of any kind of drug (MW, solubility, etc.), versatility, etc., as described elsewhere [22]. Moreover, liposomes can be designed to merge with bacterial cells, offering a potential strategy to overcome antimicrobial resistance and the formation of biofilms. This represents an encouraging approach for addressing potentially life-threatening infections caused by multidrug-resistant bacteria, such as methicillin-resistant *Staphylococci* [23]. The preparation of Dapto-loaded proliposomes for oral delivery was previously reported [24], as well as that of flexible Dapto-liposomes for skin delivery [25]. Additionally, the co-encapsulation of Dapto with clarithromycin in PEG-coated liposomes was demonstrated to enhance the antimicrobial activity against MRSA [26].

In this context, we investigate herein the development and optimization of liposomal Dapto and evaluate the antimicrobial activity of optimal Dapto-liposome types (compared to the free drug) against four Staphylococci strains. The strains were selected between 20 clinical isolates that were previously characterized and studied for their susceptibility towards Dapto; two strains were found to be less susceptible towards Dapto and two were more Dapto-susceptible; the three that were also methicillin-resistant were selected for use in the current study [16].

## 2. Materials and Methods

1,2-distearoyl-*sn*-glycerol-3-phosphatidylcholine (PC), 1,2-distearoyl-*sn*-glycero-3-phospho-(19-rac-glycerol) (sodium salt) (PG), and 1,2-Distearoyl-sn-glycerol-3-phosphatidyl-ethanolamine-N-[methoxy (polyethylene-glycol)-2000] (PEG) were purchased from Lipoid, Ludwigshafen am Rhein, Germany. Cholesterol (Chol) was purchased from Sigma–Aldrich (Darmstadt, Germany). Dapto was purchased from Tocris Bioscience. All solvents and chromatography materials used were of analytical or HPLC grade and purchased from Merck (Darmstadt, Germany). All other materials, such as salts used for buffer preparation, reagents for lipid concentration determination, etc., were of analytical grade and were purchased from Sigma–Aldrich (Darmstadt, Germany). Spectrapor^®^ dialysis membrane with an MWCO of 12–14 kDa was from Serva, Heidelberg, Germany. Materials used for antimicrobial activity evaluation are mentioned in the sections of the specific methods applied (below).

### 2.1. Preparation of Daptomycin-Loaded Liposomes

The lipid compositions used for liposome preparation were PC/Chol (1:1 mole/mole) and PC/PG/Chol (8:2:10 mole/mole/mole). After preparation, all liposome types were purified from non-encapsulated drugs using size exclusion chromatography on Sepharose 4B-CL column (1 × 30), eluted with PBS, pH 7.40. When needed, a post-PEGylation method was applied as previously reported [27]. In brief, a PEG-lipid micellar dispersion (with the required concentration) was incubated with the Dapto-loaded liposomes at 45 °C for 1 h, resulting in the integration of the PEG moieties in the outer layer of the liposome membrane.

#### 2.1.1. Thin Film Method (TFM)

Appropriate amounts of lipid solutions (according to the lipid composition) in CH_3_Cl/MeOH (2/1 *v*/*v*) for a final lipid concentration of 10 mg/mL were placed in a 100 mL round-bottomed flask. Organic solvents were evaporated under a vacuum at 41 °C using a rotary evaporator until a thin lipid film was formed, and flasks were placed under an N_2_ stream for 5 min to remove any residual traces of organic solvents. The dried lipid film was hydrated with a 250 μg/mL Dapto solution in PBS (1 mL) at 41 °C and dispersion vortexed and sonicated to form multilamellar vesicles (MLVs). Subsequent size reductions were carried out via the sequential extrusion of the MLVs (10 times) through polycarbonate filters with a pore diameter of 0.4 µm and then 0.1 µm, fitted in a syringe-type extruder (Lipo-so-fast, Avestin, Ottawa, ON, Canada), to produce SUVs. After purification (as mentioned above), liposome dispersions were concentrated using ultrafiltration (MW cutoff 10,000 daltons) to achieve the required concentration. Samples were then stored at 4 °C until further use.

#### 2.1.2. DRV Method

For dehydration–rehydration vesicle (DRV) preparation, empty small unilamellar vesicles (SUVs) were initially prepared as described in detail [28,29]. Multilamellar vesicles (MLVs) were prepared as described above (2.1.1), with the difference that the dried lipid film was hydrated using a 1 mL solution of 10% PBS at 41 °C. MLVs were then converted into SUVs using a microtip-probe sonicator (Vibra cell, Sonics and Materials, Suffolk, UK) at 26% amp for 10 min until a partially transparent solution was obtained. The next steps of the procedure are similar to the previous (thin-film hydration), including sonication, annealing (1 h at 40 °C), and centrifugation at 15,000× *g* for 20 min in order to obtain liposome suspensions (SUVs) that were free of titanium, and these were left to stand for at least 1 h at 40 °C in order to anneal any structural defects. Then, 1 mL of the SUV suspension at a lipid concentration of 10 mg/mL was mixed with 1 mL of a 250 μg/mL Dapto solution (in distilled H_2_O). The mixtures were then frozen at −80 °C for 3 h and dried under vacuum (below 5 Pa). The powder was then re-suspended initially in 100 µL of dH_2_O and incubated at RT for 30 min, which was repeated one more time, and then, finally, 800 µL of PBS was added, and the vesicle dispersion was incubated at RT for 1 h. Subsequent size reductions were carried out as described above; extrusion was used as a size-reduction method in order to prevent the disruption of the DRVs and leakage of the encapsulated drug. After extrusion, liposomes were purified (as above) from non-encapsulated Dapto.

#### 2.1.3. MicroFluidics Mixing (MM)

Liposomes were prepared using the automated Nanoassemblr platform (Precision Nanosystems, Vancouver, BC, Canada) with 2 input NxGen cartridges. The lipid composition tested was PC/Chol (1:1 mole/mole), starting with 20 mg/mL lipid in ethanol and 150 μg/mL Dapto (in PBS). Two variables can be modulated in the MM apparatus: the total flow rate (TFR) and flow rate ratio (FRR), which is defined as the volumetric ratio of the aqueous phase stream (PBS-drug) to the organic phase stream (ethanol-lipids). The flow rates of both solutions are controlled through software. Initially, the TFR was set at 12 mL/min, and the FRR was 5:1. For optimization studies, three Dapto concentrations were used, ranging between 150, and 500 μg/mL. Moreover, the FRR values tested were 1:1, 2.5:1, or 5:1, and TFR values of 4, 8, and 12 mL/min were applied. In a second round of runs, TFR values between 0.7–6 mL/min were tested.

For the removal of any solvent residuals, two rounds of ultrafiltration were performed using a molecular weight cut-off of 10,000 MWCO tubes. Liposomes were purified to remove non-encapsulated Dapto, as described above.

### 2.2. Physicochemical Characterization of Dapto-Liposomes

#### 2.2.1. Dapto Encapsulation Efficiency

Dapto concentration in liposomes was quantified using isocratic high-performance liquid chromatography (HPLC) using a Shimadzu 20A5 Gradient HPLC system coupled to aSPD-20A Prominence UV/VIS detector operating at 223 nm. An RP-18e (100 A, 5 μm, 125 × 4 mm; LiChrosphere^®^, Darmstadt, Germany) was used; the mobile phase comprised a mixture of acidified water (0.1% trifluoroacetic acid) and acetonitrile at 60:40 *v*/*v*. The column was eluted at a flow rate of 1 mL/min at 30 °C, and Dapto was eluted at 5.01 min. The sample injection volume was 50 µL. Liposomes were analyzed after being totally lysed in methanol (one volume of the sample was mixed with 10 volumes of methanol, and the mixture was agitated using a vortex). The final lipid concentration of analyzed samples was adjusted to 0.3 mg/mL. A calibration curve in the range of 0.5–40 µg/mL was constructed by preparing standard solutions of Dapto in media with similar composition as the samples (in MeOH, and in the presence of 0.3 mg/mL of lipid with composition PC/Chol 1:1 mole/mole or PC/PG/Chol 8:2:5 mole/mole/mole). Encapsulation efficiency (equivalent to drug loading capacity) was calculated using the following equation:

$$Encapsulation\;efficiency=\;\frac{\frac{D}{L}\;Final\;(\frac{mol}{mol})}{\frac{D}{L}\;Initial\;(\frac{mol}{mol})\;}\times 100$$

where *D* is drug concentration and *L* is lipid concentration; *initial* means before and *final* after purification. Liposome lipid concentration was measured routinely using the Stewart assay [30], a colorimetric method used for the quantification of phospholipids.

#### 2.2.2. Liposome Size Distribution, Zeta-Potential and Morphology

The particle size distribution (mean hydrodynamic diameter and polydispersity index (PDI)) of Dapto-loaded liposomes dispersed at 0.4 mg/mL lipid, in phosphate-buffered saline (10 mM) with a pH of 7.40, was measured using dynamic light scattering (DLS) (Malvern Nano-Zs, Malvern Instruments, Malvern, Worcestershire, UK) at 25 °C and a 173° angle [29]. Each sample was measured 11 times in three independent measurements. The polydispersity index (PDI) was used as a measure of the homogeneity of liposomal dispersions. Dispersions with a PDI of less than 0.200 or 0.250 are generally considered to have a narrow size distribution. Zeta potential was measured in the same dispersions, at 25 °C, utilizing the Doppler electrophoresis technique, as recently reported [29].

Liposome morphology was assessed using transmission electron microscopy (TEM). The liposomes (0.5–1 mg/mL) were re-suspended in 10 mM HEPES (to eliminate potential artifacts from phosphate salts). Then, samples were negatively stained with 1% phosphotungstic acid in dH_2_O (freshly prepared), washed 3 times with dH_2_O, drained with the tip of a tissue paper, and observed at 100,000 eV with a JEM-2100 (Jeol, Tokyo, Japan) transmission electron microscope.

#### 2.2.3. Drug Release and Stability Studies

A dialysis membrane method was used to follow the release kinetics of Dapto from the different liposome types. The experiment was performed at two different conditions: by keeping the initial lipid concentration or by keeping the initial drug concentration constant. Briefly, in the first case, 0.5 mL of Dapto-liposomes with 3.6 mg/mL of lipids was placed in a dialysis bag, while, in the second case, 0.5 mL of Dapto-liposomes with 12 μg/mL Dapto was used. Bags were immersed in 15 mL vials containing 15 mL PBS (pH 7.40) that were screw-capped and placed in a shaking (50 rpm) incubator at 37 °C for up to 240 h. At predetermined time intervals, the entire medium was withdrawn and replaced with a fresh one; samples were assayed using HPLC for the Dapto concentration. A calibration curve in the range of 0.00625–0.2 µg/mL was constructed by preparing standard solutions of Dapto in media with a similar composition as the samples.

The physical stability of Dapto-loaded liposomes during storage at 4 °C for up to 30 d was determined by measuring the vesicle mean diameter and PDI of the two different Dapto-liposome types as described above, immediately after their preparation as well as after 15 d and 30 d.

It is known that Dapto is degraded in solution when stored at temperatures higher than 2–8 °C [31]. Dapto chemical stability studies were carried out in order to evaluate if liposome encapsulation preserves Dapto from degradation. Free Dapto (solution in PBS) and Dapto-liposomes containing 22 ± 2.1 μg/mL of the drug were placed separately in hermetically sealed screw tubes (to avoid evaporation) and then in an orbital incubator (Stuart S1500, Fisher Scientific, Loughtborough, UK) set at 50 rpm and 37 °C for a period of up to 16 d. A second batch of samples was stored at 4 °C. At specific time intervals, samples were analyzed using HPLC, for the quantification of intact Dapto, using the method mentioned above.

### 2.3. Antimicrobial Activity (In Vitro)

#### 2.3.1. Bacterial Strains and Growth Conditions

Two well-characterized MRSA strains of *S. aureus* (71406 [MIC, 1 µg/mL] and 71221 [MIC, 0.38 µg/mL]), and two strains of *S. epidermidis* (9817 [MIC 0.75 µg/mL], which is also methicillin-resistant, and 783 [MIC 0.19 µg/mL]), were used for antimicrobial studies, selected as mentioned above [16]. All strains were grown aerobically in Tryptic soy broth (TSB, Oxoid CM0129, Oxoid Ltd., Basingstoke, UK) and on Tryptic soy agar plates (TSA, Oxoid) at 37 °C overnight. CaCl_2_-supplemented (1.25 mM) Dapto solutions were used in all antimicrobial activity studies since Dapto acts using calcium-dependent potassium efflux from bacterial cell membranes [12,13].

The zeta potential of the bacterial strains used in this study was measured using DLS, as previously described [32,33]. In brief, bacterial suspensions equivalent to the 0.5 MacFarland turbidity standard (~1.5 × 10^8^ cfu/mL) were prepared in TSB broth. The bacterial suspensions were centrifuged at 10,000 rpm for 20 min, and the supernatants were discarded. Then, cell pellets were washed five times with 0.5 mM potassium phosphate buffer solution (pH 7.4). Cell pellets were finally re-suspended in 1 mL of buffer, and zeta potential measurements were performed.

#### 2.3.2. Bacterial Growth Curve Assay

Bacterial growth was spectrophotometrically monitored [29] in the presence and absence of Dapto liposomes, empty liposomes, and mixtures of free Dapto + empty liposomes or free Dapto at two concentrations, namely, 0.5 and 1 μg/mL. Briefly, overnight-grown bacterial cells from a TSA agar plate were allowed to grow in fresh TSB broth (without glucose) to their early exponential phase. The broth containing bacteria was inoculated into 96-well flat-bottomed polystyrene plates with initial absorbance at λmax 570 nm ~0.01. The change in the absorbance of each well was monitored each hour for a total of 24 h using a Fluostar (BMG LABTECH, Ortenberg, Germany) microplate reader. A culture of the same strain without antibiotics was used as a control. Experiments were performed in triplicate.

#### 2.3.3. Biofilm Susceptibility Assays (Prevention and Treatment)

The anti-biofilm activity was evaluated at 0.1 and 0.5 μg/mL drug concentrations for all the formulations, according to previously reported methods [16,29]. Crystal violet (CV) staining assay and a validated MTT [3-(4,5-dimethylthiazol-2-yl)-2,5-diphenyltetrazolium bromide, a yellow tetrazole] cell viability assay were used to assess biofilm susceptibility towards Dapto formulations. Briefly, one single bacteria colony isolated from fresh agar plates was inoculated into a tube filled with 5 mL sterile TSB and incubated at 37 °C for 24 h. Fresh bacterial suspensions were prepared in TSB with 1% glucose from overnight cultures and adjusted to a 0.5 MacFarland turbidity standard, followed by 1:10 dilution into fresh media. Then, 200 µL of the suspension was added to 96-well sterile polystyrene plates and incubated at 37 °C for 24 h.

For *biofilm prevention studies*, antimicrobial agents were added together with bacteria.

For *biofilm reduction studies*, antimicrobial agents (Dapto formulations) were added in mature, preformed biofilms. For the later studies, following overnight incubation of bacteria in the plates, the plates were gently washed with 1× PBS (pH 7.4) to remove planktonic cells, and the well-formed biofilm was incubated with the Dapto formulations at 37 °C for 24 h.

In both (prevention and treatment study) cases, following incubations, the bacterial suspension of each well was gently spent, washed three times with PBS (pH 7.4), and stained with 195 µL of 0.1% crystal violet (Sigma–Aldrich, St. Louis, MO, USA) for 15 min at RT. Excess crystal violet was removed by washing with tap water, and biofilm was quantified by measuring the corresponding OD-570 nm of the supernatant following the solubilization of CV in 95% ethanol. For each sample (free or liposomal) tested, biofilm assays were performed in triplicate, and the mean biofilm absorbance value was determined.

In the MTT assay, biofilms were incubated with MTT (0.5 mg/mL) at 37 °C for 1 h. After washing, the purple formazan crystals that formed inside the bacterial cells were dissolved using acidified isopropanol and then measured using a microplate reader by setting the detecting and reference wavelengths at 570 nm and 630 nm, respectively.

### 2.4. Statistical Analysis

IBM SPSS statistics software was used for the statistical analysis of the results. All experiments were performed in triplicate. All data are presented as the mean ± standard deviation of the mean of independent experiments. Statistical significance was evaluated using one-way ANOVA or two-way ANOVA and LSD’s post hoc test with a significance level of *p* < 0.05.

## 3. Results

### 3.1. Dapto Liposome Physicochemical Properties

Dapto liposomes were prepared using three different methods. As seen in Table 1 and Table 2, the thin-film hydration (TFH) and DRV methods resulted in a similar encapsulation of Dapto (around 30%) for both lipid compositions tested. However, Dapto encapsulation was dramatically lower (around 4%) when liposomes were prepared using the microfluidic mixing (MM) method for both lipid compositions evaluated. Concerning Dapto liposome sizes, the vesicles prepared using the TFH and DRV methods had mean diameters of around 100 nm (102 ± 12 nm), which is logical, since they were extruded through 100 nm pore membranes, while the liposomes prepared with MM had a similar size in the case of PC/Chol composition but a smaller size when PG was included in the liposome membrane (Table 2).

The zeta potential values of Dapto-liposomes composed of PC/Chol were slightly negative, ranging between −3.7 and −8.8 (Table 1). Liposome types that encapsulated higher amounts of Dapto (TFH and DRV) had higher negative zeta-potential values compared to the MM liposomes that encapsulated an approximately eight times lower amount of Dapto. The latter observation is logical since Dapto, as an anionic lipopeptide antibiotic, bears a negative charge at pH 7.4. When 20 mol% of PC is replaced with a negatively charged PG lipid (Table 2), the charge of the lipid determines the liposome zeta potential, which is around −20 mV for all the liposome types (TFH, DRV, MM), irrespective of the amount of Dapto they incorporate.

As seen from the results in Table 3, when PEG is included in the lipid composition of liposomes, the encapsulation of Dapto is dramatically reduced. Such decreased drug loading into PEG liposomes has been reported before. In one case, for vinorelbine loading, Chol-polyethylene glycol was used instead of PEG for the preparation of polyethylene glycol-coated liposomes with high drug loading [34]. In our laboratory, we also observed a similar reduction in drug loading into PEGylated liposomes, especially when using the DRV method, for relaxin peptide and moxifloxacin [27,29], and found that the post-PEGylation method could be applied for the preparation of PEGylated liposomes with high drug loading. In agreement with the previous cases, it was possible to apply the post-pegylation procedure on pre-formed, Dapto-loaded liposomes by incubating the liposomes with PEG micelles for 1 h at 45 °C. Indeed, the Dapto liposomes retained most if not all of their Dapto content, and no significant effect on their other physicochemical properties was conferred using post-PEGylation, with the exception of the high drop of the negative zeta potential value of the PG-containing Dapto-loaded liposomes that occurred after PEGylation (from −30.9 to −13.4), which, in fact, proves that the negatively charged lipid containing the membrane of the vesicles was successfully coated with the hydrophilic polymer chains, in agreement with other reports [27,29]. A similar drop in negative zeta potential was also observed when PEGylation was applied by adding the lipid-polymer conjugate in the lipid phase of the PG-containing liposomes (from −30.9 to −8.7), proving that PEGylation was achieved; however, in that case, Dapto loading was highly reduced. Overall, PEGylated Dapto-loaded liposomes with high Dapto loading could be achieved for non-charged and negative charge liposomes using post-PEGylation.

Due to the very low EE (%) of Dapto in liposomes prepared using MM, we decided to use the liposomes prepared using the TFH method for the remaining studies. The MM method was further explored in order to investigate if, by modulating the mixing parameters, or by using different organic solvents and/or initial drug concentrations, higher Dapto amounts could be encapsulated in the liposomes; however, increased Dapto loading in nanosized vesicles could not be achieved. Given the unique structure of this acidic lipopeptide drug, it will be interesting to further explore the MM preparation of Dapto liposomes by designing experiment approaches. Nevertheless, the high encapsulation efficiency (EE) found in several preparations (>30%) using TFH and DRV methods (Table 1, Table 2 and Table 3) can be explained in view of the peculiar interaction of Dapto with the liposome bilayer, and not by its encapsulation within the aqueous core, for which much lower EE is expected [35]. Perhaps the MM method does not succeed in attaining a high encapsulation for Dapto because the drug is directed to the aqueous core of the vesicle during rapid liposome formation.

The physical stability of liposomal Dapto formulations composed of PC/Chol (1:1) and PC/PG/Chol (8:2:10), and prepared using the TFH, DRV, or MM methods, was additionally studied, as mentioned in the Methods section. Experimental results indicate that, for both liposome membrane compositions tested, the liposomes prepared using the TFH and DRV methods demonstrated high physical stability (mean diameter and PDI values did not change) for the one-month period that was evaluated; oppositely, a gradual slight increase in the vesicle mean diameter was observed in the case of the liposomes prepared using the MM method (Supplementary Figure S1).

### 3.2. Liposomal Dapto Release and Chemical Stability Studies

**Figure 1:**
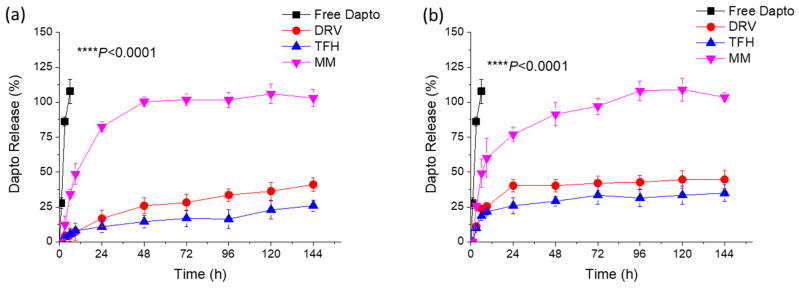
Release of Dapto from PC/Chol (1:1) (**a**) or PC/PG/Chol (8:2:10) (**b**) formulated using the TFH, DRV, or MM method. The sample drug concentration was adjusted at 12 μg/mL. Results are expressed as a percentage of the initial drug for each sample. Experiments were carried out in triplicate; mean values ± SD are reported. Two-way ANOVA *p* values of liposome-type effect are reported.

**Figure 2:**
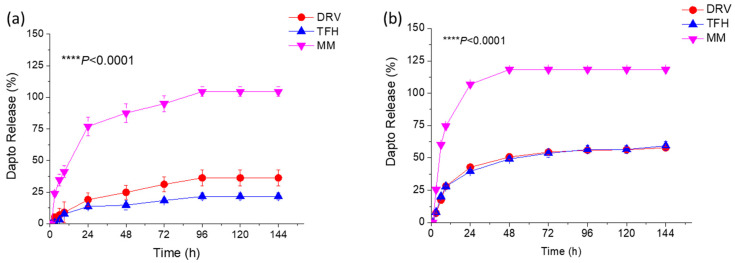
Release of Dapto from liposomes consisted of (**a**) PC/Chol (1:1) and (**b**) PC/PG/Chol (8:2:10) formulated using the TFH, DRV, or MM methods. The sample lipid concentration was 3.6 mg/mL. Results are expressed as the percentage of the initial drug for each sample. Experiments were carried out in triplicate; mean values ± SD are reported. Two-way ANOVA *p* values of liposome-type effect are reported.

The release of Dapto from the different types of liposome compositions (Table 1 and Table 2) was studied under two conditions, either by keeping the drug concentration in the samples constant (Figure 1) or by keeping the liposomal lipid concentration constant (Figure 2) at 37 °C.

In both cases, sink conditions were applied throughout the experiments. As seen, Dapto was released much faster from the liposomes prepared using the MM method, compared to the other two methods, regardless of the lipid composition. Dapto-liposomes prepared using the TFH and DRV methods had similar Dapto-releasing profiles for both lipid compositions studied; however, Dapto released from the MM liposomes was significantly faster (for both liposome types). It is thus indicated that the very low amount of Dapto encapsulated in the ΜΜ liposomes is perhaps more loosely associated with the liposomes (or a different mode of association of the lipopeptide with the MM liposomes exists), an observation that requires further exploration. In any case, the longer retention of Dapto in the DRV and THF liposomes is another reason for selecting the TFH method for the following experiments, since we have recently observed substantially higher antimicrobial activity in moxifloxacin liposomes compared to other liposome types from which the release of antibiotics was slower (compared to other liposomes that released the drug faster) [29].

Since it is known that Dapto is degraded in solution, we sought to investigate if perhaps liposomal encapsulation can provide protection toward Dapto degradation, as previously reported for other molecules, such as relaxin and curcumin [27,36]. Thereby, in another set of experiments, the stability of liposomal Dapto was evaluated during incubation at 37 °C or 4 °C for up to 16 days.

**Figure 3:**
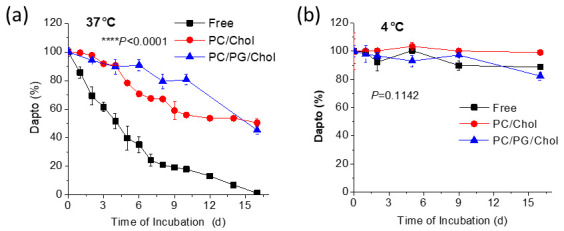
Kinetics of liposomal and free Dapto (solution) degradation during incubation at (**a**) 37 °C and (**b**) 4 °C for up to 16 d. Results are expressed as a percentage of the initial drug for each sample. Experiments were carried out in triplicate; mean values ± SD are reported. Two-way ANOVA *p* values of formulation-type effect are reported.

From the results reported in Figure 3a, it becomes evident that liposomal encapsulation protects Dapto from degradation during incubation in aqueous media at 37 °C. Indeed, free Dapto is degraded rapidly (Figure 3a), resulting in more than 30% degradation after 48 h; meanwhile, after 16 days of incubation at 37 °C, no free Dapto is detected. Oppositely, under the same incubation conditions, no degradation of liposomal Dapto is observed after 48 h, and more than 45% of the initial amount of liposomal Dapto is detected (in both liposomes evaluated) after 16 days. On the contrary, Dapto is stable, even as a free drug solution when incubated at 4 °C for the full period evaluated (Figure 3b). These results prompted us to evaluate if the antimicrobial properties of liposomal Dapto were correspondingly retained for longer periods at 37 °C, and a separate study was carried out, as mentioned below, in Section 3.5.

### 3.3. Transmission Electron Microscopy of Dapto Liposomes

**Figure 4:**
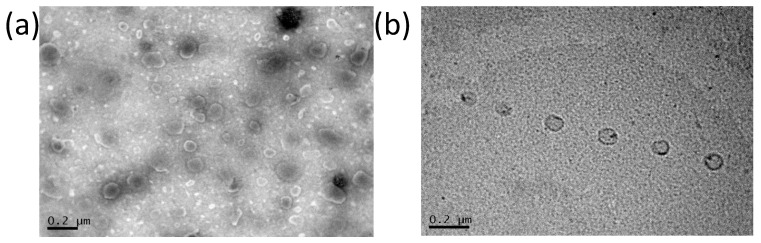
TEM micrographs of Dapto liposomes: (**a**) PC/Chol (1:1); (**b**) PC/PG/Chol (8:2:10).

Transmission electron microscopy was performed to complete the characterization of Dapto liposomes. As seen in Figure 4, both types of liposomes are round-shaped, and the vesicle diameters observed in TEM micrographs (around 100 nm) agree with the DLS measurements in Table 1, Table 2 and Table 3.

### 3.4. Inhibition of Planktonic Bacterial Growth by Dapto Liposomes-Effect of Lipid Composition

**Figure 5:**
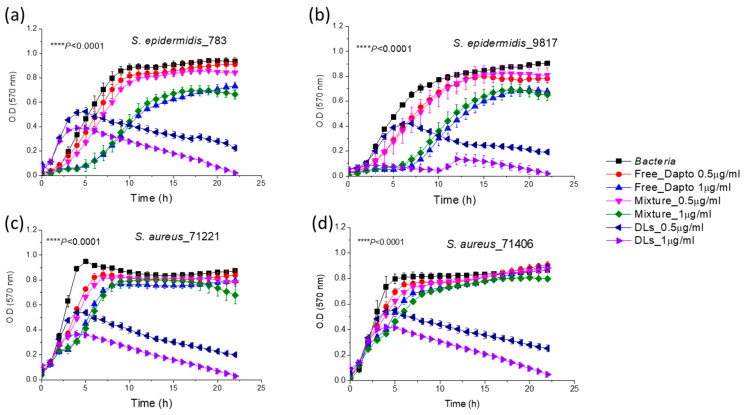
Growth curves of *S. epidermidis* 783 (**a**) and 9817 (**b**), as well as 71221 (**c**) and 71404 (**d**) in the presence and absence of 0.5 µg/mL (0.308 μM) and 1 μg/mL (0.616 μM) Dapto as free or liposomal drug. Mixtures of empty liposomes (94.7 and 189.6 μM lipid) with free Dapto were also used as controls; the lipid composition of the liposomal formulations applied was PC/Chol (1:1).

As shown in Figure 5, the growth of all four bacterial strains of *S. epidermidis* and *S. aureus* bacteria was substantially inhibited by PC/Chol Dapto-liposomes (DLs) at both drug doses (0.5 and 1 µg/mL) tested. Liposome formulations could inhibit bacterial growth significantly better compared to the same concentration of the free drug in all cases. In fact, the bacteria count in the samples that were incubated for 24 h in the presence of a high dose of liposomal Dapto was below 2% (of the corresponding initial count) for all the bacterial strains studied, indicating very high bacteriostatic activity of PC/Chol liposomal Dapto. On the other hand, empty liposomes (with the same lipid composition and at the lipid concentrations (94.7 and 189.6 µM lipid) corresponding to the two liposomal drug doses used did not have any significant inhibitory effect on bacterial growth for any of the bacteria (not shown in Figure 4 for increased clarity). Finally, the mixtures of the free drug (0.5 and 1 µg/mL) and empty liposomes (94.7 and 189.6 µM lipid, respectively) conferred (in all cases) similar effects on bacterial growth as that of the free drug. The latter observation indicates the importance of the association of Dapto with the PC/Chol liposomes (and not just mixing the two components together) for the inhibition of bacterial growth.

**Figure 6:**
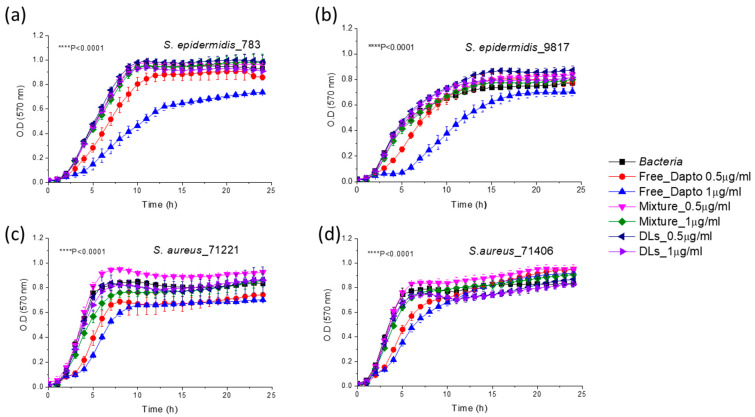
Growth curves of *S. epidermidis* 783 (**a**) and 9817 (**b**), as well as 71221 (**c**) and 71404 (**d**) in the presence and absence of 0.5 µg/mL (0.308 μM) and 1 μg/mL (0.616 μM) Dapto as free or liposomal drug. Mixtures of empty liposomes (94.7 and 189.6 μM lipid) with free Dapto were also used as controls; the lipid composition of the liposomal formulations applied was PC/PG/Chol (8:2:10).

Surprisingly, when Dapto is encapsulated in PC/PG/Chol liposomes, the activity of liposomal Dapto against all the species of planktonic bacteria is diminished; no significant growth inhibition is demonstrated by liposomal Dapto for any of the bacterial strains tested (Figure 6).

The main difference between the two liposomal Dapto formulations is the much higher negative charge of the PC/PG/Chol liposomes (−30.9 ± 1.6) (Figure 6) compared to the PC/Chol liposomes (−8.8 ± 2.3) (Figure 5), as reported in Table 3. We thereby hypothesize that perhaps the PC/PG/Chol liposomal Dapto does not demonstrate any inhibitory effect on the planktonic bacteria due to electrostatic repulsion between the suspended bacteria and liposome vesicles. This hypothesis is strengthened by the negative zeta potentials measured as described in the methods section for all the bacterial strains used in growth inhibition studies (Table 4).

Similar effects of liposome surface charge on liposomal antibiotic inhibitory action towards planktonic bacteria have been reported before. It has been recently reported that negatively charged gentamicin-loaded liposomes exhibited the same bacteriostatic concentration as that of free gentamicin, while the minimum bactericidal concentration of neutral gentamicin-loaded liposomes towards planktonic *P. aeruginosa* bacteria was twofold lower than that of free gentamicin [37]. Similar enhanced antimicrobial activity of liposomal gentamycin was also reported elsewhere [38]. The different bacteriostatic activities of the two liposome types (neutral and negative) were attributed to the “limitation of liposomal fusion with a negatively charged bacterial cell wall due to repulsive forces at close proximity”, in accordance with our suggestion. Similar results were also found by others, and it was concluded that cationic liposomes could interact more with the negatively charged outer membrane of Gram-negative cells or, in the case of Gram-positive bacteria, with the thick peptidoglycan cell wall compared to anionic and neutral liposomes [39].

### 3.5. Antibiofilm Activity of Dapto Liposomes—Effect of Lipid Composition

**Figure 7:**
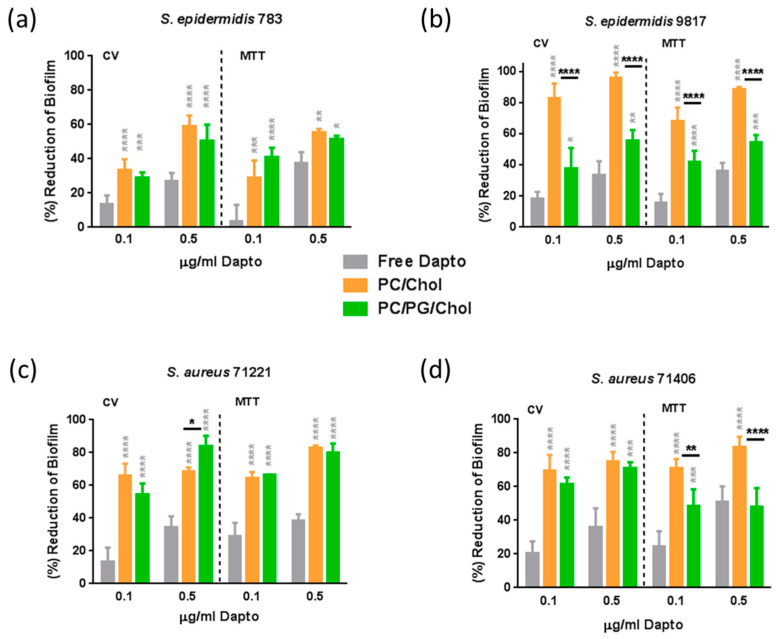
Biofilm prevention studies: Reduction (% compared to untreated control) of biofilm mass (CV) and biofilm bacteria viability (MTT) of (**a**) *S. epidermidis* 783, (**b**) *S. epidermidis* 9817, (**c**) *S. aureus* 71221, and (**d**) *S. aureus* 71406 using Dapto solution (Free) and two types of liposomal Dapto (PC/Chol and PC/PG/Chol) at doses of 0.1 and 0.5 µg/mL. The significance of the difference from free is presented as grey asterisks on top of corresponding bars and separately black asterisks denote significant differences between liposome types. *: *p* ≤ 0.05; **: *p* ≤ 0.01, ***: *p* ≤ 0.001; ****: *p* ≤ 0.0001.

**Figure 8:**
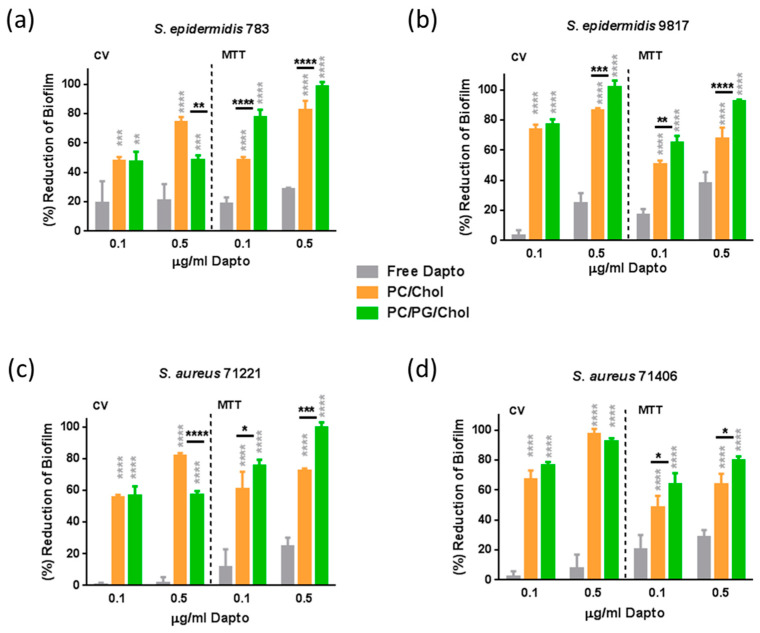
Biofilm treatment studies: Reduction (% compared to untreated control) of biofilm mass (CV) and biofilm bacteria viability (MTT) of (**a**) *S. epidermidis* 783, (**b**) *S. epidermidis* 9817, (**c**) *S. aureus* 71221, and (**d**) *S. aureus* 71406 using Dapto solution (free) and two types of liposomal Dapto (PC/Chol and PC/PG/Chol) at doses of 0.1 and 0.5 µg/mL. The significance of difference from free is presented as grey asterisks on top of the corresponding bars and separately black asterisks denote significant differences between liposome types. *: *p* ≤ 0.05; **: *p* ≤ 0.01, ***: *p* ≤ 0.001; ****: *p* ≤ 0.0001.

The effect of liposome lipid composition on the anti-biofilm activity of Dapto was also studied by employing bacterial biofilm susceptibility assays with the same bacterial strains and the same liposomal preparations as those used for bacterial growth inhibition studies. Dapto concentrations of 0.1 µg/mL (0.061 μM) and 0.5 μg/mL (0.308 μM), and corresponding lipid concentrations of 18.9 μM and 94.7 μM for PC/Chol liposomes, and slightly lower 16.3 μM and 81.6 μM for PC/PG/Chol liposomes (due to their slightly higher encapsulation efficiency (Table 4)) were used. Two sets of experiments were carried out, biofilm prevention experiments (where therapeutics were incubated with the bacteria before biofilm formation) and biofilm treatment experiments (where therapeutics were incubated with pre-formed biofilms). The biofilm prevention study results are presented in Figure 7, and the results of the biofilm treatment studies are seen in Figure 8. In both cases, the results are expressed as the percent of the reduction in biofilm mass (CV) and the percent of the reduction in biofilm bacterial viability (MTT).

From Figure 7, it is observed that free daptomycin has significant activity against the biofilms (compared to untreated samples) for all of the bacteria tested, especially at the highest dose used. Indeed, when the bacteria biofilm is formed in presence of 0.5 μg/mL free Dapto, both the mass of the biofilm and biofilm (bacteria) viability are reduced from 27.5% to 36.2% (mass) and from 36.4% to 51.1% (viability) compared to biofilms that formed in absence of Dapto, in accordance with previous studies that report the higher activity of Dapto towards biofilm bacteria compared to planktonic bacteria [9,16]. Moreover, similar biofilm reductions were observed towards all the bacterial strains tested, namely, the more susceptible bacteria, *S. epidermidis* 783 and *S. aureus* 71221 (Figure 7a and Figure 7c, respectively), and the more resistant strains, *S. epidermidis 9817* and *S. aureus 71406* (Figure 7b and Figure 7d, respectively), proving the potential of liposomal Dapto also towards biofilms of resistant bacterial strains.

Nevertheless, biofilm mass (CV) as well as biofilm viability values were reduced several times more when Dapto-loaded liposomes were incubated with the bacteria compared to the corresponding reductions conferred by free Dapto. Dapto liposomes (both lipid compositions tested) demonstrated dramatically higher biofilm prevention activity compared to free Dapto towards all the bacterial strains evaluated. Indeed, when the bacterial biofilms are formed in the presence of 0.5 μg/mL liposomal Dapto, both the mass of the biofilm and the biofilm (bacteria) viability are reduced from 59.5% to 96.2% (mass) and from 55.7% to 88.1% (viability) by PC/Chol liposomes and from 50.2% to 83.8% (mass) and from 48.1% to 80.3% (viability) by PC/PG/Chol liposomes (compared to biofilms that formed in the absence of Dapto). Especially at the lower Dapto concentration studied (0.1 µg/mL), where the reductions conferred by the free drug (in biofilm mass and viability) were <30% for all bacteria, liposomal Dapto formulations conferred a reduction in biofilm mass between 1.9 and 7.5 times higher and between 1.6 and 6.3 times higher reduction in biofilm viability compared to the free drug.

Even more interesting is the fact that very high reductions in biofilm masses and viabilities are conferred by liposomal Dapto towards the resistant strains *S. epidermidis* 9817 and *S. aureus* 71406. In fact, the Dapto liposomes demonstrated the lowest biofilm prevention activity towards the less resistant *S. epidermidis* 783 strain (when comparing the biofilm reductions conferred towards the different bacterial strains tested), which is also the only methicillin-susceptible strain.

From Figure 7, it is also evident that, in some cases, significant differences in the Dapto-liposome-conferred biofilm prevention activity are noticed between the two types (lipid compositions) of liposomes used in the study. In fact, PC/Chol liposomes demonstrate the highest activities, the only exception being the slightly higher (and marginally statistically significant) reduction in *S. aureus* 71221 bacteria biofilm mass by PC/PG/Chol liposomes. It may be postulated that, since, in the biofilm prevention studies, the liposomes are mixed with bacteria before the formation of the biofilm, any initial electrostatic repulsion between the negatively charged bacteria and the PC/PG/Chol liposomes may result in fewer liposomes being integrated into the biofilms, reducing the action of these liposomes.

As seen in Figure 8, in established biofilms, the anti-biofilm effect of free Dapto is lower compared to that observed in the biofilm prevention studies (Figure 7). Indeed, when the bacteria biofilm is formed in the presence of 0.5 μg/mL free Dapto, both the mass of the biofilm and the biofilm (bacteria) viability are reduced from 1.5% to 24.6.% (mass) and from 24.8% to 38.1% (viability) compared to biofilms that formed in the absence of Dapto. This is logical since pre-established mature biofilms are much more difficult to treat with antibiotics, posing a well-known unmet medical need.

However, the biofilm reduction activities demonstrated by liposomal Dapto were surprisingly very high, suggesting that Dapto liposomes may be considered for the treatment of resistant biofilms. Indeed, as depicted in Figure 8 results, Dapto liposomes (both lipid compositions tested) demonstrated dramatically higher biofilm reduction activity compared to free Dapto towards all the bacterial strains evaluated. Indeed, when the pre-formed bacterial biofilms are treated with 0.5 μg/mL liposomal Dapto, both the mass of the biofilm and the biofilm (bacteria) vitality are dramatically reduced (eradicated in some cases) from 74.6% to 97.6% (mass) and from 63.7% to 82.2% (viability) by PC/Chol liposomes and from 48.4% to 100% (mass) and from 80.2% to 100% (viability) by PC/PG/Chol liposomes (compared to biofilms that formed in absence of Dapto). At the lower Dapto concentration studied (0.1 µg/mL), where the reductions conferred by the free drug (in biofilm mass and viability) were <20% for all bacteria, liposomal Dapto formulations conferred a reduction in biofilm mass between 2.6 and 73 times higher and a reduction in biofilm viability between 2.6 and 6.6 times higher compared to free drug.

Furthermore, very high anti-biofilm activity is conferred by liposomal Dapto towards all bacterial strains evaluated, particularly towards the more resistant strains *S. epidermidis* 9817 (Figure 8b) and *S. aureus* 71406 (Figure 8d), for which the high liposomal Dapto dose resulted in a complete eradication of the pre-formed biofilm masses. In fact, the Dapto liposomes demonstrated the lowest biofilm prevention activity towards the less resistant *S. epidermidis* 783 strain (when comparing the biofilm reduction conferred towards all the strains tested). Another factor that should be considered is that the action of Dapto liposomes would be expected to be higher on moderate biofilm-producing bacteria due to their biofilm structure, which may better allow liposomal penetration. As seen in Table 5, the *S. epidermidis 783* strain produced the biofilms that had the highest mass and viability compared to all the other strains (in both cases); therefore, we can consider that this strain is a stronger biofilm producer compared to the other three (which could be considered as moderate biofilm producers). In fact, the efficacy of liposomes to reduce the biofilm mass of the three latter strains (*S. aureus* 71406, *S. aureus* 71221, *S. epidermidis* 9817—moderate biofilm producers) is higher compared to their efficacy in reducing the biofilm mass of the *S. epidermidis* 783 strain (Figure 7 and Figure 8), indicating that, perhaps, the penetration into the biofilm of the stronger biofilm-producing strain is more difficult, which is in agreement to the above assumption.

It should be clarified at this point that empty liposomes (PC/Chol and PC/PG/Chol) at similar concentrations as those used for Dapto liposomes were also evaluated for potential biofilm prevention and treatment and did not demonstrate any significant difference regarding biofilm mass and viability compared to untreated biofilms (see Supplementary Figure S2). This overrules the possibility that the anti-biofilm activity of Dapto liposomes may be partly attributed to the prevention of bacteria adhesion (due to the presence of liposomes) during biofilm production. In fact, by comparing the liposome-induced reduction values of biofilm mass with the corresponding reduction in biofilm viability, in Figure 7 and Figure 8, it is evident that, if not completely, at least to a high percent, the anti-biofilm effect of Dapto-liposomes is due to biofilm bactericidal activity.

Another very interesting observation from the results of the biofilm treatment study (Figure 8) is that, in contrast, to what was seen in the biofilm prevention studies, here, in most cases, the PC/PG/Chol negative charge liposomes demonstrated the highest activities, with the exceptions of a biofilm mass reduction in (less resistant) *S. epidermidis* 783 (Figure 8a) and *S. aureus* 71221 (Figure 8c) bacteria (where PC/Chol liposomes acted better).

Perhaps PC/PG/Chol liposomes allow better penetration into some biofilms since, in the biofilm treatment studies, the drug formulations added on are already formed biofilms and, therefore, the charge of the biofilm components and not the charge of the bacteria should be more important. In fact, similar results were previously reported regarding the effect of a liposome charge for the treatment of bacterial biofilms. In one study, negatively charged clarithromycin-loaded liposomes were reported to have increased activity against a *P. aeruginosa* biofilm [40]. In another study, negatively charged tobramycin-loaded liposomes were observed to be immobilized close to a biofilm cluster due to the electrostatic attraction between the cluster and the liposomes. This led to the penetration of the liposomes into the biofilm and to subsequent bacteria killing [41]. Additionally, negatively charged gentamycin-loaded liposomes exhibited higher anti-biofilm activity against *P. aeruginosa* and *K. oxytoca* compared to the free drug but also to neutral liposomes [39].

### 3.6. Preservation of Antibiofilm Activity of Dapto by Liposome Encapsulation

**Figure 9:**
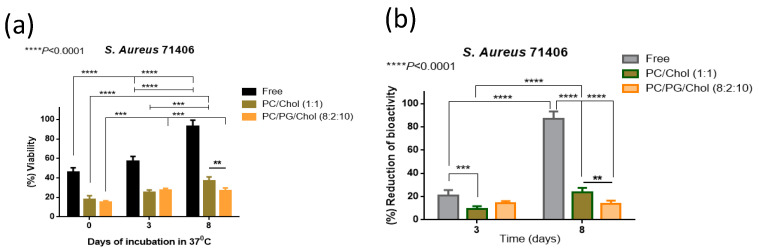
(**a**) Bioactivity (expressed as cell viability of *S. aureus* 71406 biofilm after treatment with 0.5 μg/mL of free or liposomal Dapto formulations, following their incubation for various durations at 37 °C) and (**b**) bioactivity reduction (expressed as reduction of bioactivity from the initial value at time 0). **** *p* ≤ 0.0001; *** *p* ≤ 0.001 ** *p* ≤ 0.01.

As mentioned above, we proved that the liposomal encapsulation of Dapto protects the drug from chemical degradation (Figure 3), and a question was posed regarding the antimicrobial activity of free and liposomal Dapto following the subjection of Dapto formulations at 37 °C that take place after in vivo administration. For this, an additional experiment was carried out to measure the bioactivity of free and liposomal Dapto formulations after incubation at 37 °C for 3 d and 8 d. More specifically, the reduction in the viability of the *S. aureus* 71406 pre-formed biofilm was assessed using the same methodologies used for the biofilm treatment studies (Figure 9).

As seen in Figure 9b, the reduction in the bioactivity of Dapto after 3 and 8 days is highest for free Dapto (21%) and similar for the two liposome types at day 3 (approx. 10%). At day 8, most of the bioactivity of free Dapto (~80%) is lost, while PC/Chol liposomes lose significantly more bioactivity compared to PC/PG/Chol liposomes. The later results correlate well with the Dapto degradation study results (Figure 3a), suggesting that the bioactivity of Dapto is reduced linearly with its integrity. From the current results, it is proven that liposomal encapsulation preserves not only the chemical integrity but also the biofilm reduction activity of Dapto liposomes, suggesting that the higher activity of liposomal Dapto (compared to the free drug) is at least partly attributed to the protection provided by liposomal encapsulation; of course, this is not the only mechanism involved, as already discussed before.

## 4. Discussion

Summarizing the findings of the current report, three methods were used for the formation of nanosized Dapto-encapsulating liposomes. THF and DRV methods showed similar Dapto loading ability and sustained release of the drug from the liposomes, while MM methods could not provide liposomes with similar EE% and a release profile of Dapto, although their size and other properties were similar; further investigations are needed for an elucidation of the involved mechanisms.

Concerning the antibacterial activity of Dapto liposomes, neutral charge liposomes conferred significantly increased bacteriostatic activity (compared to the free drug) towards the planktonic bacteria of two *S. epidermidis* and two *S. aureus* clinical strains (most being methicillin-resistant), while negative charge liposomes had no activity, in agreement with previous reports in the relevant literature for other liposomal drugs [37,38,39]. Furthermore, biofilm prevention and biofilm treatment studies revealed the high potential of Dapto liposomes to reduce biofilm mass and viability towards all the bacterial strains studied, compared to free Dapto. Interestingly the charge of liposomes seemed to determine their antibiofilm activity in an opposite way to that demonstrated for their bacteriostatic activity against planktonic bacteria, a phenomenon also reported before for other drugs [39,40,41]. Thus, the previous theories to explain the effect of liposomal antibiotic surface charge on their inhibitory activity towards planktonic bacteria growth, as well as on their biofilm treatment efficacy, are further strengthened.

Dapto integrity and bioactivity preservation studies during the incubation of free and liposomal drugs at 37 °C showed that liposome encapsulation protects the lipopeptide drug from chemical degradation and preserves its bioactivity in a similar manner, providing one potential mechanism for the higher potency of liposomal Dapto compared to the free drug. We cannot be sure which other mechanisms previously proposed to explain the high potency of other liposomal antibiotics, such as the fusion between liposome and bacterial cells that results in antibiotic direct delivery in bacterial cytoplasm [42] and/or the enhanced penetration of liposomes into bacterial biofilms [41], which are implicated in the current findings; however, the current results provide another example about the high potential of liposomal antimicrobials.

Especially for daptomycin, which is a particular drug with a large cyclic lipopeptide and good activity to reduce persistent and methicillin-resistant biofilms related to medical devise infections, simple liposome formulations such as those studies herein could find applications as potent therapeutic solutions for treating persistent biofilms; additionally, liposomal Dapto integration into medical devices could be considered [43].

In fact, in two studies concerning daptomycin liposomes reported before, targeted liposomes were considered, in which Dapto was conjugated with PEG and attached on a liposome surface to assist their targeting to bacteria and also loaded alone or with another drug in the liposomes [44,45]. In the earlier study, such Dapto liposomes demonstrated specific binding to MRSA using flow cytometry and good targeting capabilities in vivo to MRSA-infected lungs in a pneumonia model [44]. In the second, more recent report, the targeted liposomes were also loaded with Vancomycin and were additionally coated with erythrocyte ghosts, being thus a particularly complicated formulation. In comparison to free drugs, the formulations sustained the release of drugs for 3 days and evaded detection by macrophages. Additionally, the targeted liposomes reduced the MIC and significantly increased bacterial permeability (compared to the free drug), resulting in more than 80% bacterial death within 4 h [45]. Other previous studies involving Dapto liposomes included (i) proliposomes that were prepared for oral delivery and were found to realize a significant increase in oral bioavailability of Dapto [24]; (ii) flexible Dapto-liposomes that were found to enhance the ability of Dapto to permeate the skin and demonstrate antibacterial activity against biofilms [25]; (iii) liposomes for the co-delivery of Dapto with clarithromycin that were demonstrated to produced significant anti-MRSA activity in the presence of only one-thirtieth of the concentration required when only Dapto-liposomes were used [26]. Due to the fact that different types of liposomes were formulated in the previous studies (with different sizes, lipid compositions, and initial drug/lipid ratios (for liposome preparation)), we cannot directly compare them with our Dapto liposomes; however, the antimicrobial activities reported are well correlated with the current findings.

In light of the current findings that planktonic bacteria, as well as the matured biofilms of MRSA and MRSE bacterial strains (that also have low susceptibility towards Dapto), were almost completely eradicated by the liposomal Dapto formulations developed herein. Perhaps the use of more simple Dapto liposomes alone or in combination with other antimicrobials should be considered in designing novel antimicrobial therapeutic systems against medical device-associated infections. Such simple liposomal Dapto formulations have the additional benefit of being more easily translatable into drug products.

## Figures and Tables

**Table 1:** Dapto-liposome (PC/Chol (1:1 mol/mol)) EE (%), mean diameter, PDI, and zeta potential. TFH, DRV, and MM methods were used. Each value is the mean of three different samples ± the corresponding SD of each mean.

Method	EE (%)	Mean Hydr. Diameter (nm)	PDI	ζ-Pot (mV)
**TFH**	27.9 ± 1.8	119.2 ± 6.5	0.198	−8.7 ± 2.3
**DRV**	31.7 ± 4.0	102.6 ± 5.7	0.068	−8.80 ± 0.17
**MM**	8.1 ± 1.0	113.8 ± 7.6	0.282	−3.7 ± 1.4

**Table 2:** Dapto-liposome (PC/PG/Chol 8:2:5 mol/mol/mol) EE (%), mean diameter, PDI, and zeta potential. TFH, DRV, and MM methods were used. Each value is the mean of three different samples ± the corresponding SD of each mean.

Method	EE (%)	Mean Hydr. Diameter (nm)	PDI	ζ-Pot (mV)
**TFH**	30.1 ± 6.6	98.3 ± 6.3	0.065	−23.2 ± 1.1
**DRV**	37.6 ± 7.3	103.5 ± 1.77	0.046	−21.9 ± 1.8
**MM**	4.2 ± 0.5	83.8 ± 4.4	0.192	−20.5 ± 2.3

**Table 3:** Properties of Dapto liposomes (TFH) with different lipid compositions. Each value reported is the mean of three different samples and the corresponding SD of each mean is reported.

Lipid Composition	EE (% D/L)	Mean Hydr. Diameter (nm)	PDI	ζ-Potential (mV)
PC/Chol (2:1)	25.8 ± 5.8	122.2 ± 9.5	0.93	−6.2 ± 2.9
PC/Chol (1:1)	30.06 ± 5.2	119.23 ± 6.48	0.198	−8.8 ± 2.3
PC/Chol/PEG (1:1:0.17)	3.56 ± 0.009	117.1 ± 8.4	0.125	−7.7 ± 0.6
PC/PG/Chol (8:2:5)	37.2 ± 9	127 ± 12	0.182	−21.60 ± 0.75
PC/PG/Chol (8:2:10)	**31.2 ± 5**	**120.3 ± 9.3**	**0.182**	**−30.9 ± 1.6**
PC/PG/Chol/PEG (8:2:10:1.7)	3.56 ± 0.21	98.6 ± 5.3	0.187	−8.7 ± 1.0
PC/Chol/PEG (1:1:0.17) Post-PEG *	25.6 ± 2.0	106.7 ± 6.3	0.159	−8.93 ± 0.47
PC/PG/Chol/PEG (8:2:10:1.7) Post-PEG *	**32.2 ± 1.5**	**138.7 ± 8.8**	**0.175**	**−13.4 ± 3.0**

* post-PEGylation of pre-formed liposomes without PEG.

**Table 4:** Zeta potential values of planktonic bacteria. Each value reported is the mean of three different samples, and the corresponding SD of each mean is reported. Post-PEG stands for post-PEGylation of pre-formed liposomes without PEG.

Bacterial Strain	ζ-Potential (mV)
*S. epidermidis* _783	−28.7 ± 1.30
*S. epidermidis* _9817	−20.8 ± 1.48
*S. aureus* _71221	−11.7 ± 3.44
*S. aureus* _71406	−23.8 ± 1.88

**Table 5:** CV and MTT staining OD-570 nm values of the control bacteria incubated as mentioned in the Methods section, for biofilm prevention and biofilm reduction studies. Each value reported is the mean of three different samples, and the corresponding SD of each mean is reported.

Bacterial Strain	Biofilm Prevention Studies		Biofilm Reduction Studies	
	CV	MTT	CV	MTT
*S. epidermidis* 783	1.6 ± 0.03	0.9 ± 0.07	2.3 ± 0.09	1.5 ± 0.04
*S. epidermidis* 9817	0.32 ± 0.04	0.3 ± 0.08	0.8 ± 0.09	0.5 ± 0.09
*S. aureus* 71406	0.6 ± 0.1	0.8 ± 0.08	2.1 ± 0.1	0.8 ± 0.1
*S. aureus* 71221	0.4 ± 0.2	0.4 ± 0.08	1.7 ± 0.02	0.9 ± 0.1

## Data Availability

Dataset available on request from the authors.

## Supplementary Materials

The following supporting information can be downloaded at: https://www.mdpi.com/article/10.3390/pharmaceutics16040459/s1, Figure S1: Physical stability of Dapto liposomes; Figure S2: Control study to evaluate empty liposome effect on the prevention and treatment of bacterial biofilms.
