# Supplementary material for: Daptomycin Liposomes Exhibit Enhanced Activity against Staphylococci Biofilms Compared to Free Drug
Source: Pharmaceutics. 2024 Mar 26;16(4):459. doi: 10.3390/pharmaceutics16040459 (PMC11054717; doi:10.3390/pharmaceutics16040459)
Supplement: Supplementary file 1 [file pharmaceutics-16-00459-s001.zip › Supplementary Figure S1.pdf]

Supplementary Figure S1: Physical stability of Dapto liposomes

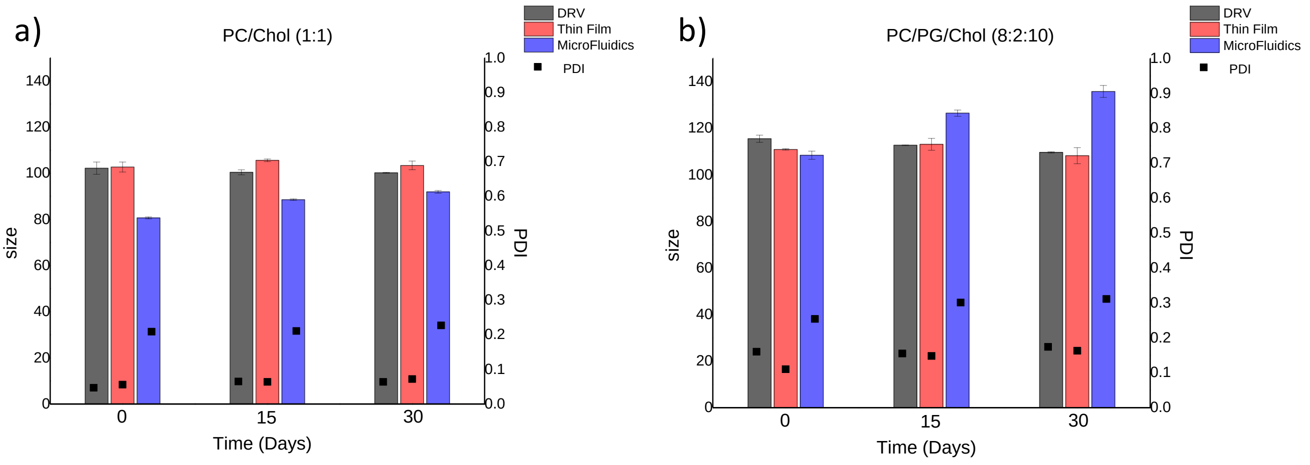

Figure S1. Mean hydrodynamic diameter (nm) and PDI values of Dapto liposomes, after preparation and after storage at 4°C for 15 d and 30 d. **a)** PC/Chol (1:1, mol/mol) liposomes; **b)** PC/PG/Chol (8:2:10, mol/mol) liposomes.
