# Supplementary material for: Daptomycin Liposomes Exhibit Enhanced Activity against Staphylococci Biofilms Compared to Free Drug
Source: Pharmaceutics. 2024 Mar 26;16(4):459. doi: 10.3390/pharmaceutics16040459 (PMC11054717; doi:10.3390/pharmaceutics16040459)
Supplement: Supplementary file 1 [file pharmaceutics-16-00459-s001.zip › Supplementary Figure S2.pdf]

Supplementary Figure S2: Control study to evaluate Empty Liposome effect on Prevention and Treatment of Bacterial Biofilms

Figure S2-1:Biofilm Prevention Study results

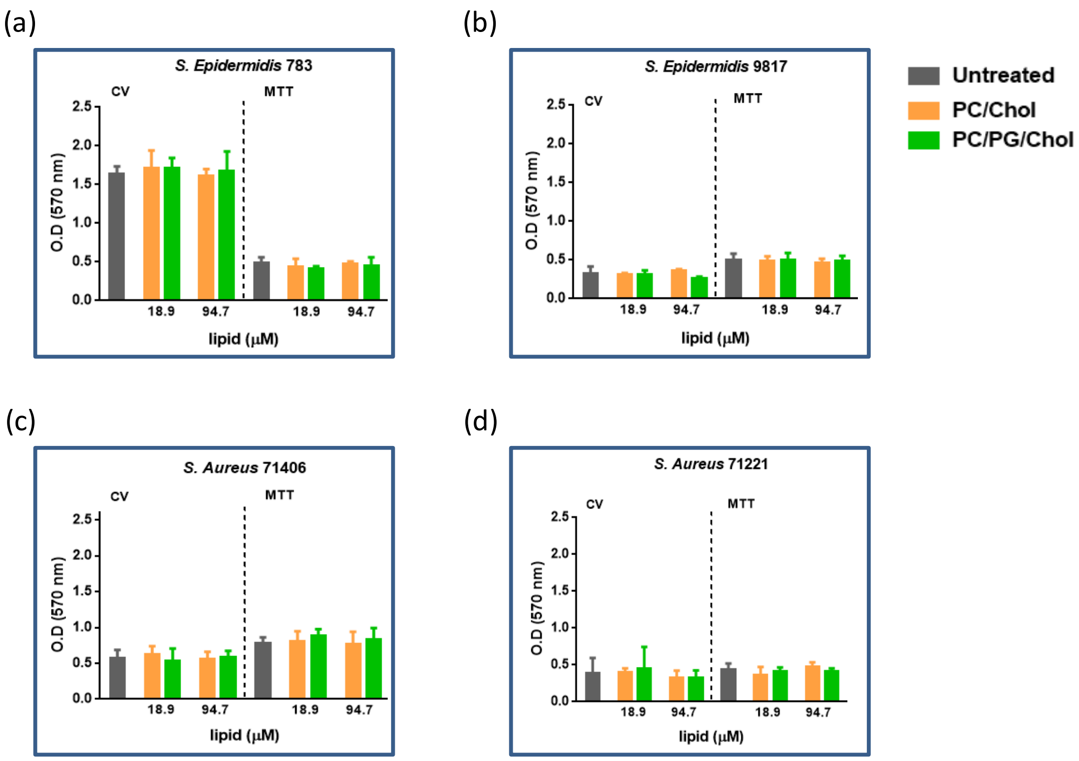

Figure S2-1. OD-values of empty liposomes and untreated biofilms, for biofilm mass (CV) and biofilm bacteria viability (MTT) of (a) *S.epidermidis* 783, (b) *S.epidermidis* 9817, (c) *S. aureus* 71406 and (d) *S. aureus* 71221, by empty liposomes at the same concentrations used in the Dapto liposome biofilm prevention experiments of Figure 7. No Significance differences were detected between empty liposome values and corresponding untreated values, for any liposome type (composition)

Figure S2-2:Biofilm Treatment Study results

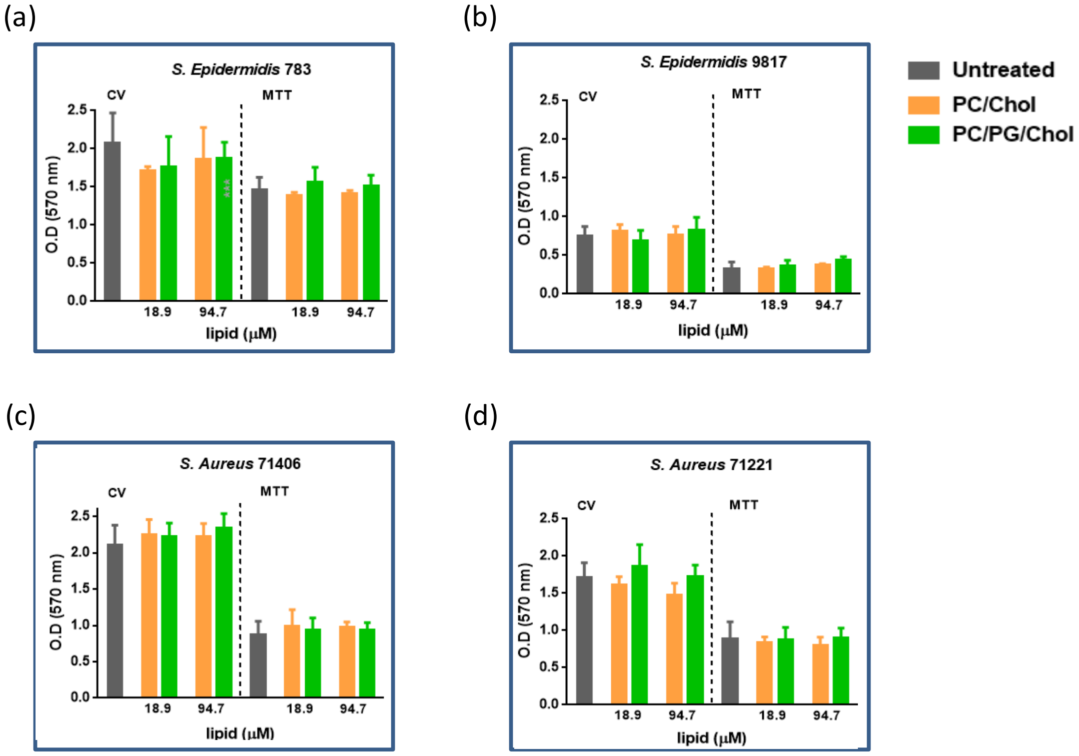

Figure S2-2. OD-values of empty liposomes and untreated biofilms, for biofilm mass (CV) and biofilm bacteria viability (MTT) of (a) *S.epidermidis* 783, (b) *S.epidermidis* 9817, (c) *S. aureus* 71406 and (d) *S. aureus* 71221, by empty liposomes at the same concentrations used in the Dapto liposome biofilm prevention experiments of Figure 8. No Significance differences were detected between empty liposome values and corresponding untreated values, for any liposome type (composition)
